# Persistence Assessment of Chemicals: Trajectories toward New Approach Methodologies (P-NAMs)

**DOI:** 10.1021/acs.est.6c00444

**Published:** 2026-04-08

**Authors:** Kathrin Fenner, Daniel Zahn, Ulrich Jöhncke, Gabriel Sigmund, Philipp Mayer, Christopher Hughes, Hans Peter H. Arp, Russell J Davenport, Anu Kapanen, Thorsten Reemtsma

**Affiliations:** † 30964 Eawag, Swiss Federal Institute of Aquatic Science and Technology, Dübendorf 8600, Switzerland; ‡ Department of Chemistry, University of Zürich, Zürich 8057, Switzerland; § 28342 Helmholtz Centre for Environmental Research − UFZ, Department of Environmental Analytical Chemistry, Leipzig 04318, Germany; ∥ German Environment Agency, Section IV 2.3 Chemicals, Dessau-Roßlau 06844, Germany; ⊥ Environmental Technology, 4508 Wageningen University, Wageningen 6700 AA, Netherlands; # Department of Environmental and Resource Engineering, Technical University of Denmark, Kongens Lyngby 2800, Denmark; ∇ Embark Chemical Consulting Ltd, Chester, CH2 1LR, U.K.; ○ 72989 Norwegian Geotechnical Institute (NGI), Oslo 0484, Norway; ◆ Norwegian University of Science and Technology (NTNU), Trondheim 7491, Norway; ¶ School of Engineering, Newcastle University, Newcastle Upon Tyne, NE1 7RU, U.K.; △ 138826 European Chemicals Agency, Helsinki 00121, Finland; ▲ Faculty of Chemistry, University of Leipzig, Leipzig 04103, Germany

**Keywords:** biodegradation kinetics, ready biodegradability, simulation studies, REACH, high-throughput testing, transformation products, biodegradation models

## Abstract

Chemical persistence has long been recognized as a critical determinant of ecosystem and human exposure, exemplified by legacy pollutants such as DDT, PCBs, and, more recently, PFAS. Despite decades of regulation and research, robust experimental half-life data are available for only a fraction of chemicals in use, hampering their persistence assessment. Current testing frameworks, while refined, lack the efficacy to address these large data gaps, underscoring the need for innovative approaches. We argue that new approach methodologies for persistence assessment (P-NAMs)—including high-throughput (HT) experimental systems and advanced *in silico* models—are needed. HT-testing can bridge the gap between biodegradability screening tests and resource-intensive simulation studies. Simultaneously, HT-testing can generate large, consistent data sets needed to improve the mechanistic understanding of biotransformation and train more accurate predictive models. Integration of transformation product analysis and FAIR (findable, accessible, interoperable, and reusable) data repositories will further enhance mechanistic understanding and model reliability. We call for coordinated efforts across academia, industry, and regulatory bodies to establish standardized reporting, expand accessible data sets, and validate predictive tools. By advancing P-NAMs, the scientific community can ensure that persistence assessment evolves from a regulatory bottleneck into a driver of innovation, safeguarding human and ecosystem health and promoting safe chemical design.

## Graphical abstract

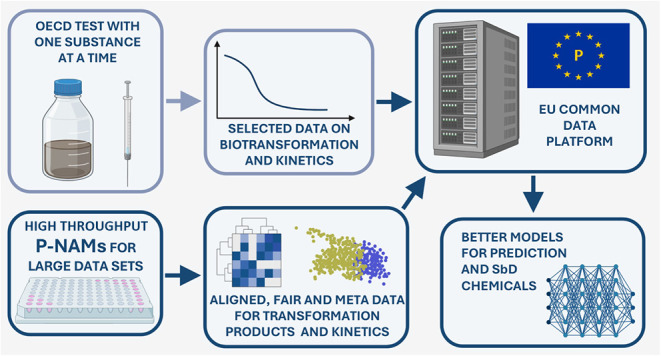

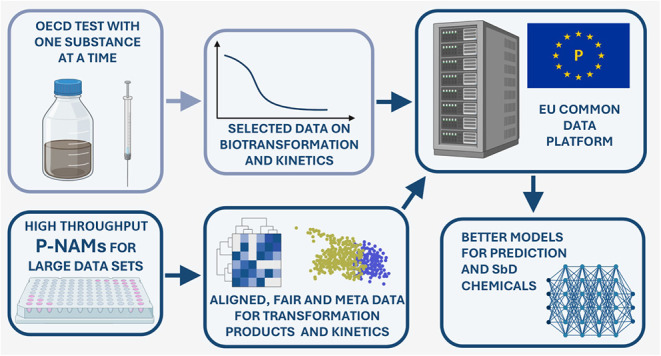

## Introduction

Persistent substances are those with a low potential to degrade under environmental or standard laboratory conditions.^1^ Concern over man-made persistent substances dates back to dichlorodiphenyltrichloroethane (DDT), polychlorinated biphenyls (PCBs), and dioxins, and was fueled more recently by the ubiquitous detection of extremely persistent perfluorinated alkyl substances (PFAS).^2^ These cases confirmed that persistence is a key driver of ecosystem and human exposure,^3^ particularly for mobile and/or bioaccumulative substances. At the same time, for some applications, persistence is a necessary chemical property to fulfill a desired function.

Following regulations in the early 1960s on poorly degradable surfactants, persistence has increasingly become recognized as a key hazard criterion in chemical regulations worldwide.^4^ Consequently, it was incorporated into the Stockholm Convention on Persistent Organic Pollutants, adopted in 2001, currently including 186 ratifying parties and adhering jurisdictions. Persistence is also considered in the REACH regulation (EC 1907/2006) and, more recently, the European Regulation on Classification, Labeling and Packaging of Chemicals (CLP; EC 1272/2008 and EU 2023/707). The UN Globally Harmonized System of Classification and Labeling of Chemicals (UN-GHS) does not currently include criteria for persistence, but considers lack of rapid degradability as part of the chronic aquatic toxicity hazard identification.^5^ To support safer chemicals, voluntary frameworks like the Green Chemistry principles^6^ and the EU’s Safe-and-Sustainable-by-Design (SSbD) framework^7^ explicitly call for degradability. Consequently, knowledge on persistence of chemicals is crucial to adequately assess and manage the hazards and risks of chemicals as part of regulatory frameworks, and to select and develop chemicals that align with SSbD and green chemistry principles for future societal needs.

Despite decades of research and regulation, knowledge on persistence for the hazard and risk assessment of existing chemicals and for the development and provision of safer, new chemicals is still insufficient.^8^ For REACH registered substances, biodegradation “screening tests” such as ready biodegradability (e.g., OECD 301 A–F, OECD 310) and inherent biodegradability (OECD 302 B, C) have historically been the focus and approach typically used for assessing the degradability of chemicals. Data from these tests are thus expected to be available, where required. However, many substances fail the stringent criteria of these tests, and, under the current regulatory frameworks, require “simulation tests” generating half-life data in order to conclude on persistence. Furthermore, for some substances, such as those of unknown or variable composition (UVCBs), screening tests are not fit for purpose. Between 2016 and 2020, more than 400 requests for further information on biodegradation were issued under REACH processes.^9^ Yet, according to a 2020 assessment by Arp and Hale,^8^ half-lives were available for 292 unique substances only, roughly 2.2% of the 13 405 REACH substances and transformation products under consideration. This leaves large parts of REACH registered substances with insufficient data for a robust persistence assessment, in part due to the highly time- and resource-consuming data generation with simulation tests.

Accordingly, the European Chemicals Agency (ECHA) highlighted the need for new tools that allow for accelerating persistence assessment in their 2025 report on key areas of regulatory challenge (KARC).^10^ Specifically, the report highlights: (i) the need for more high-throughput testing methods for identifying (non)­persistent substances; (ii) the need for “middle level tests” that bridge screening and higher tier tests; and (iii) that these data should be used to develop *in silico* tools that can predict degradation rates in relevant environmental media.

As scientists from academia, regulation and private companies active in the field of persistence assessment, we agree with ECHA’s analysis. We further emphasize that the present status of persistence assessment does not only challenge regulation, but also environmental sciences and chemicals innovation. In this Perspective article, we elaborate on how to address these challenges, alongside a more specific aspect—the formation of transformation products. We argue for a new trajectory toward New Approach Methodologies (NAMs) for Persistence assessment (P-NAMs) (Figure 1), and postulate that P-NAMs, including high-throughput tests and *in silico* models for persistence assessment, are instrumental in two ways:

- First, high-throughput tests can serve as a bridge between stringent ready biodegradability screening tests and the more environmentally relevant but highly time- and resource-consuming biodegradation simulation studies, and, in doing so, support decision-making in industrial and regulatory contexts (Figure 1, vertical arrow).
- Second, high-throughput tests allow for the generation of larger and better aligned biodegradation data sets. These data sets will foster the understanding of the structural and environmental determinants of biodegradability, and enable the training of more accurate *in silico* models across a wider chemical space (Figure 1, horizontal arrow).

**Figure 1:**
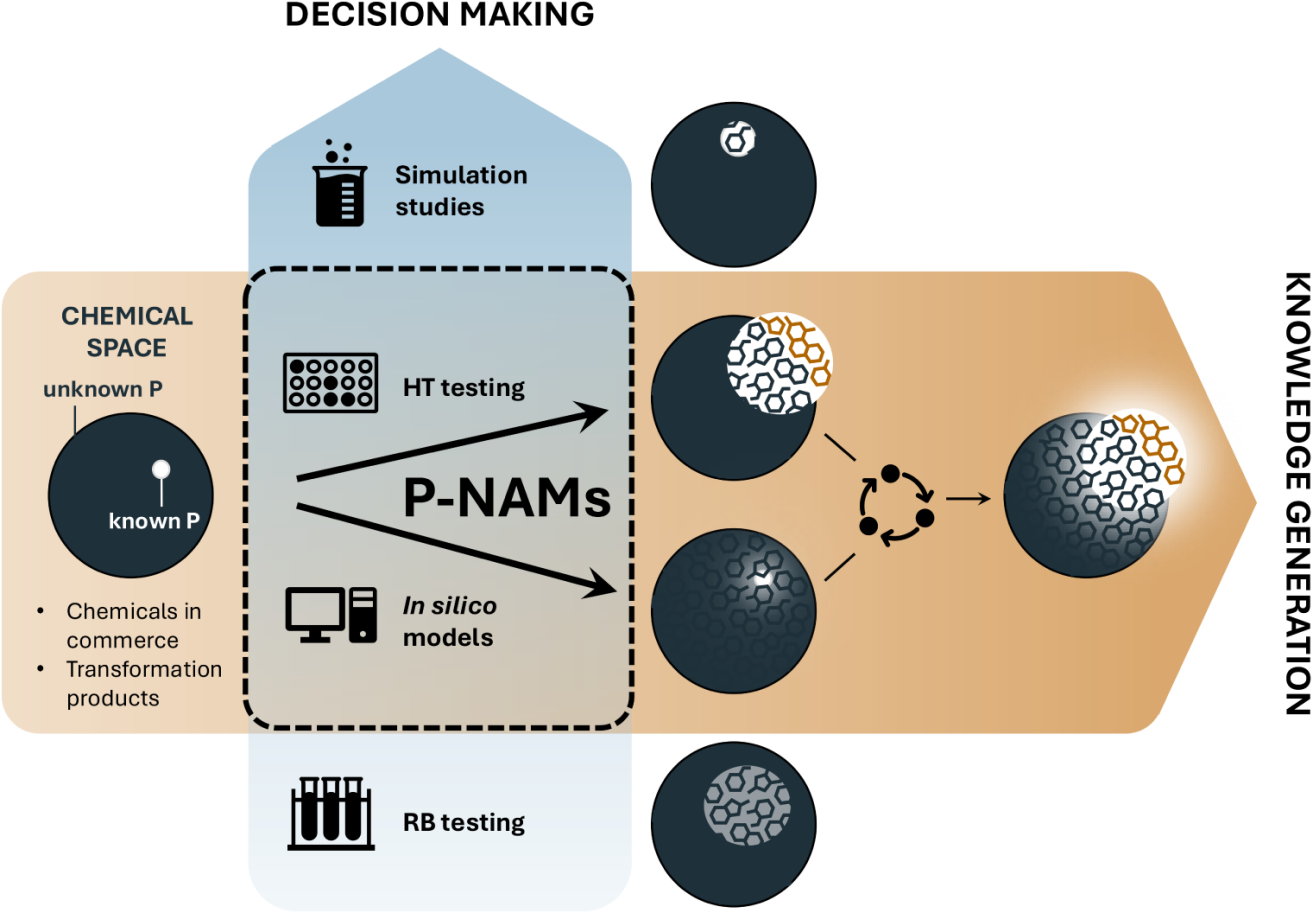
New approach methodologies for persistence assessment (P-NAMs), i.e., high-throughput (HT) biodegradation tests and *in silico* models for predicting persistence, will bridge the gap between ready biodegradability (RB) testing and simulation studies and thus support decision making in regulatory and industrial, safe-by-design contexts (vertical arrow, blue). They will also help increase knowledge on underlying drivers and dependence on molecular structure (horizontal arrow, brown). At the interface of increased data volumes, improved mechanistic understanding and *in silico* models, more accurate models that cover larger chemical spaces will eventually become tangible. Black circles represent the entire chemical space, highlighted areas represent chemical space covered by biodegradation data, shades of highlighted areas indicate information content of biodegradation data (gray—low information content, white—higher information content).

The revised Recommendation of the European Commission on an “assessment framework for ‘safe and sustainable by design’ chemicals and materials” emphasizes the imperative for research organizations to engage “in the development, promotion and uptake of new assessment methods, models and tools that can be integrated into the SSbD Framework”.^11^ High-throughput methods for biodegradation testing and more accurate *in silico* models trained with increased and consistent data volumes generated by such high-throughput tests will together serve as crucial tools to identify biodegradable and persistent structures during hazard identification at the early and intermediate stages of the innovation process. As such, P-NAMs have a high potential to advance the implementation of green chemistry and safe-by-design principles in product development.

## The Road to Better *In Silico* Models Starts with Data

*In silico* tools, or quantitative structure–biodegradation relationships (QSBR), hold promise to learn from existing biodegradation data to predict persistence for chemicals that lack experimental data. Currently, QSBRs can be used only as part of weight of evidence in persistence assessments under REACH and are considered especially useful in combination with grouping and read-across approaches for structurally similar substances.^12^ QSBRs could more efficiently support product development pipelines, where computational approaches are common in screening and early development stages.^13^

Models predicting yes/no classification outcomes of ready and inherent biodegradability tests have been developed since the 1990s, typically claiming balanced classification accuracies, i.e., the average percentage of correctly identified readily and nonreadily biodegradable substances, of 80–90%.^14^ These models are also increasingly made available as user-friendly webapps with batch prediction and explicit indication of the applicability domain (e.g., https://biodegradability-prediction-app.streamlit.app).^14,15^ It could be assumed that with increased training data sets from 250+ to over 6000 substances over the years,^14^ models have become more accurate for a wider range of chemical structures. However, this notion is challenged by a recent evaluation of several of those state-of-the-art models against an independent validation data set of 2684 chemicals compiled from the REACH registration database.^16^ This study found a balanced accuracy of the assessed models of approximately 75% only, with one of the oldest models (BIOWIN 6) performing best overall, suggesting that integration of expert knowledge via manually curated descriptors is key for the development of models that generalize well.

Models to predict environmental compartment-specific half-lives aligned with simulation test outcomes are substantially fewer. The most prominent ones include the VEGA suite of models,^17^ and most recently the PEPPER webapp (https://pepper-app.streamlit.app/) for removal in wastewater treatment plants and degradation half-lives in soil.^18,19^ Others are specific to hydrocarbons only.^20^ Despite having been developed using state-of-the-art approaches, these models testify to the fact that the “promise of artificial intelligence” is challenged for predicting environmental persistence. More specifically, while the ambition of these models is to be globally applicable across a wide range of chemicals, they de facto provide confident and accurate predictions only for a very limited part of the chemical space, i.e., the center space of the training range (Figure 1). This is largely due to the very sparse coverage of the structural space of commercial chemicals with existing half-life data—typically under 1000 substances per environmental compartment.^18,21^ Moreover, observed degradation half-lives are as much a product of chemical structure and its intrinsic degradability, as of the experimental conditions and environmental inoculum sampled. Degradation half-life data for one chemical are therefore variable, which translates into—often ignored—uncertainty in the data used for model training, and challenges the ability of modeling algorithms to uncover the structure-dependence of biotransformation half-lives (see refs 19,22 for the example of pesticide half-lives in soil and how Bayesian statistics can support explicit consideration of variability in data evaluation and model development). More standardized information, i.e., meta-data, on test conditions (inocula characteristics, temperature, test methodology) would be a prerequisite for better model calibration.

Therefore, to develop better models for predicting biodegradation half-lives and mechanisms, we first and foremost need more data and meta-data to become available, meaning:

- more in number and diversity, *i.e*., experimental data for larger numbers of chemicals that better covers the structural space of commercial chemicals,
- more consistency, *i.e*., more data generated with the same well-characterized inoculum and under the same experimental conditions to focus on the influence of chemical structure on biodegradability,
- more complementarity, *i.e*., more data for the same chemicals under varying, well characterized experimental conditions to elucidate their influence on half-lives and obtain a more accurate estimate of irreducible variability in biodegradation data,
- more transparency and consistency, *i.e*., provision of meta-data on (experimental) conditions (including inoculum concentration and characteristics) under which half-life data were generated,
- more information on both kinetics and transformation pathways, with a special focus on persistent transformation products.

We see three key strategies to tackle this. *First*, we, as a scientific community, collect existing data on biotransformation kinetics and pathways available from regulatory dossiers and scientific literature to make them electronically accessible and hence available for model development. *Second*, we ensure through journal standards and reporting requirements of funding organizations that the authors of future biodegradation studies provide results and information on experimental conditions in a FAIR format. And, *third*, we develop high-throughput experimental methods that allow for biodegradation testing of large numbers of chemicals under consistent sets of conditions with well-characterized inocula. The latter is discussed in more detail in the following section.

Besides the lack of data, challenges in model development include finding suitable structural representations for predicting biotransformation end points and learning from small and highly variable data sets. We emphasize that proper validation and reporting for any new models according to OECD QSAR model reporting formats (OECD no. 69 and 386) should become a minimum standard to support the use of QSBRs in industrial and regulatory contexts. Those hosting large collections of biodegradation data (*e.g*., the enviPath packages (https://envipath.org/package) containing biotransformation kinetics and pathway data for soil, water-sediment and activated sludge environments;^23^ FAIR-TPs (https://fairtps.lcsb.uni.lu), which can be used to explore and export published transformation and metabolite data^24^) can support benchmarking by offering standardized data splits for model development and evaluation.^25^

## Not “More of the Same” but New Test Methods

After decades of regulation on persistence and the parallel development of biodegradation tests and testing hierarchies by regulatory bodies (e.g., ECHA^1,26^ ) and standardization bodies (e.g., OECD^27^), a refined framework for persistence assessment has emerged.^4^ Efforts for further improvement are ongoing, e.g., for ready biodegradability screening and OECD 309.^28–31^ However, we cannot fill the large gap in biodegradation data within this framework alone. New, high-throughput (HT) approaches for biodegradation testing that reduce experimental effort in terms of time and costs by at least 1 order of magnitude compared to established test systems are needed to create the necessary test capacity to progress. For decision-making in regulation and industry (vertical arrow in Figure 1), higher test capacities will aid in satisfying the need for more data to address existing regulatory gaps and provide efficient screening at early product development stages to support safe-by-design principles. Higher test capacities will also allow exploring the effect of molecular structure, test conditions and inocula on observed half-lives and biotransformation pathways; this will support knowledge generation (horizontal arrow in Figure 1) by improving mechanistic understanding of the processes influencing biodegradation.

Higher throughput in biodegradation testing can be achieved by a variety of measures, alone or in combination, including scaling down test volumes, automating sample processing, simpler read-outs, shorter durations afforded by higher biomass-to-substrate ratios and testing in mixtures. Down-scaling to well-plate format and milliliter to microliter test volumes requires lower amounts of test substance and inoculum, supporting multiplexing. Such test designs align with automation infrastructure established in industry R&D for testing of target functionality and safety, and could also be adapted to mechanistic HT assays, such as enzyme assays.^32^ Shorter test durations allow more tests to be conducted in the same time. In addition, testing of chemicals in mixtures rather than one-by-one drastically increases throughput without further test modifications.

**Table 1:** Overview of Recent HT Biodegradation Testing of Chemicals

Aim	Detection system	Specific test characteristics	Compounds covered	Implications	Reference
Ready biodegradability screening test	Colorimetric detection of aromatic test compounds	96-well plate format	29 phenol and naphthol substances	Proof of principle for HT miniaturized biodegradation screening test in a 96-well format for persistence screening and prioritization using benchmark chemicals.	^36^
Ready biodegradability screening test	Colorimetric detection of 4-nitrophenol	96-well plate format	4-nitrophenol	Standard inocula preparations reduce bacterial diversity and increase reliability of results.	^33^
Ready biodegradability screening test	Microbial growth measured by flow cytometry	96-well plate format	Aniline, sodium benzoate and caffeine	Proof of principle for using microbial growth detection as a HT method for biodegradability screening.	^37^
Modified OECD 301-type ready biodegradability test for UVCBs	SPME-GC-MS combined with O_2_ optosensors	Microvolume dosing of gastight test system	UVCBs with volatile and hydrophobic constituents	Simultaneous determination of UVCB mineralization and primary degradation of multiple constituents.	^38^
Biodegradation screening in different environmental inocula	O_2_ optosensor	24-well plate format, different environmental inocula, different concentrations and temperatures	Sodium benzoate, 4-nitrophenol, diethylene glycol, 2,4,5-trichlorophenol, atrazine, and glyphosate	Probabilistic assessment across different environmental conditions (ProbaBio concept) provides robust assessment of overall biodegradation potential of a substance.	^35^
Primary biotransformation in modified OECD 309-type simulation studies	SPME-GC-MS	Primary degradation determined from peak area ratios between biotic and abiotic vials	Various chemicals tested individually and in mixtures.	No confounding mixture effects observed at low concentrations.	^39^
Primary biotransformation in modified OECD 309-type simulation studies	RPLC-HRMS	3 Swedish rivers (OECD 309 with sediment added), mixture spike	56–80 water-relevant substances (pharmaceuticals, pesticides)	No confounding mixture effects observed at low spike concentrations (*i.e*., 0.5 μg/L).	^40^
Primary biotransformation in modified OECD 309-type simulation studies	RPLC-HRMS	18 different surface waters (OECD 309 with sediment added), mixture spike	>90 structurally diverse, water-relevant substances (pharmaceuticals, agrochemicals, cosmetics, food additives, and industrial chemicals)	Quantified primary biotransformation rate constants and extent of their variability across European surface waters.	^41^
Primary biotransformation in modified OECD 309-type simulation studies	Automated SPME-GC-MS on unopened test systems	Chemicals tested in mixtures in 3 types of water	53 petrochemicals, C8–C20, 11 structural classes. Wide polarity and volatility range.	Biodegradation kinetics of chemicals in mixtures at low concentrations (ng/L-μg/L).	^42^
Primary biotransformation in activated sludge	RPLC-HRMS	3-day, 50 mL, mixture spike	>150 structurally diverse, water-relevant substances (pharmaceuticals, pesticides, and sweeteners)	Quantified primary biotransformation rate constants and extent of their variability across six WWTP under three different operation conditions (C-elimination, nitrifying/denitrifying, MBBR).	^43^
Primary biotransformation in activated sludge; Comparison well-plates with large volumes	RPLC-HRMS	24-well plate, 2 mL format and 50 mL large-volume controls, mixture spike	33 pharmaceuticals and pesticides	Biotransformation rate constants from 24-well plate format agree with large volume controls.	^44^
Primary biotransformation in activated sludge; Read-across to OECD 307 and 308 data	RPLC-HRMS	3-day, 50 mL, mixture spike	>80 pharmaceuticals and pesticides	Read-across models calibrated to use primary biotransformation rate constants from activated sludge studies to predict half-lives from OECD 307 and 308 simulation tests.	^45,46^

All these modifications, however, bring about uncertainties and limitations. Down-scaling and shortening affect microbial community size, dynamics and adaptation phenomena in an unknown way relative to existing testing protocols.^33^ Well-plate formats raise challenges such as increased gas exchange, which affects the recording of oxygen consumption or CO_2_ evolution, and likely increase numbers of “difficult to test” substances, as decreasing volumes will increase sorption and volatilization. Testing chemicals in mixtures, finally, is not compatible with read-outs like oxygen consumption or CO_2_ evolution but requires specific and sensitive, mostly mass spectrometry-based methods, which may not always be available. Besides that, the concentration and combination of chemicals in mixtures may affect the microbial community and its degradation activity, and, thus, the test outcome.

Previously published, downscaled HT biodegradation tests mostly focus on OECD 301-type ready biodegradability tests and either use optosensors for dissolved O_2_ ^34,35^ or colorimetric assays to demonstrate mineralization or degradation of specific functional groups, e.g., aromatics^36^ (Table 1). Independent of the optical read-out, these studies all demonstrated that low biomass:substrate ratios led to highly variable test outcomes, while higher ratios gave more consistent test results. To the best of our knowledge, similar HT ready biodegradability tests are offered by some contract research organizations or used internally at some companies.

For decision making with respect to regulatory persistence assessment (Figure 1, vertical arrow), ECHA highlighted the need for the development of “middle level” tests sitting between lower tier screening methods and higher tier simulation studies. These tests should be less stringent and thus more environmentally relevant than screening tests while being less complex and lengthy than simulation tests.^10^ To achieve these goals, we believe that methods using environmentally relevant inocula and biomass:substrate ratios, yet providing higher throughput than simulation tests, are needed. Two major modifications discussed in this context, relative to simulation studies, include testing chemicals in mixtures at low concentrations and reducing use of ^14^C-labeled substances to avoid costly and time-consuming synthesis of ^14^C-labeled material. “Middle level” HT-testing also holds a lot of promise in knowledge generation (Figure 1, horizontal arrow) because reduced efforts and consistent test conditions across large sets of substances allow exploring biodegradation on a much wider scale with respect to both, the structural and the environmental determinants of biodegradability.

First studies of HT “middle level” testing show promise (Table 1): (i) Smaller test volumes have been shown to yield robust test outcomes provided that the biomass density is high enough to circumvent the “bacterial lottery” problem and biomass:substrate ratios are close to environmentally relevant levels; (ii) degradation in spiked mixtures was consistent with single spike experiments at environmentally realistic concentrations; (iii) tests with mixtures of chemicals in activated sludge could provide outcomes that are reasonably predictive of half-lives from OECD 307 and 308 simulation tests.

That said, more aspects warrant consideration on the way to implementing HT methods more broadly. First, all of these new approaches should be tested on more substances and across different laboratories for further consolidation. Second, as biodegradation data will be generated more rapidly under nonstandardized conditions, specific validation strategies will be needed to ensure confidence in HT-test outcomes and clarify how they relate to standardized, simulation study outcomes. Such efforts might profit from using a selection of benchmark chemicals covering the full range of biodegradability.^47,48^ Third, more HT-testing is useless without accompanying improved data processing workflows. While colorimetric read-outs fit that bill, more high-throughput processing of mass spectrometric data including robust peak integration, automated outlier detection and smart pooling schemes for transformation product analysis as demonstrated by Gulde et al.^49^ are required to efficiently support testing in mixtures. Throughout, detailed information on the composition of a substance remains key for successful biodegradation testing, also with HT-testing. At the same time, more experience with testing of spiked mixtures might also pave the way for persistence assessment of complex substances such as UVCBs.^50^

Given the importance of biodegradation data for the regulation of chemicals, discussion of how the outcome of new tests can be used for different regulatory needs is necessary. For example, it should be considered when comparability with existing tests is a must (direct replacement of OECD standard tests or attribution of their weight-of-evidence in regulatory persistence assessments), and when not (e.g., to rank chemicals in an R&D context, to understand structure–function relationships, to screen for very persistent chemicals).

The need to improve and speed up identification of persistent substances will undoubtedly accelerate progress in HT biodegradation testing. More experience and data need to be generated to pave the way for optimal use in both decision-making and knowledge generation (Figure 1). Researchers are encouraged to share experiences, codesign lists of benchmark chemicals and cooperate to achieve faster progress on a broader scale.

## P without Transformation Products is Only Half of the Picture

The tenth principle of green chemistry^6^ (published in 1998) clearly raised the point that transformation of the parent chemical—often termed “primary (bio-)­transformation”—can lead to the formation of hazardous TPs. Indeed, an evaluation of biodegradation that omits TPs may be incomplete, yet the integration of TPs into HT-testing and prediction models brings additional challenges.^51^ So far, most HT-testing approaches focus on primary biotransformation or mineralization, and either do not specifically consider TPs, or, in some cases, cannot feasibly integrate their consideration due to detection methods and end points chosen. High-resolution mass spectrometry offers the most seamless integration of TPs but analytical challenges remain. Most TPs are more polar than their respective precursors, thus many TPs may fall outside the analytical window of widely used reversed-phase liquid chromatography (RPLC) and gas chromatography (GC) methods and thus remain overlooked even when TPs are considered. Advances in chromatographic methods for very polar chemicals, like supercritical fluid chromatography, ion chromatography and hydrophilic interaction chromatography, can complement RPLC and GC to widen the analytical window.^52^

Additionally, TP identification (“structural annotation”) is complex, time-consuming, and of limited certainty if no reference material is available. A selection of benchmark chemicals with known biotransformation products can support quality assurance with respect to their formation as well as the detection and identification of TPs. In any case, much of the effort in HT-biodegradation testing will shift from experimental work toward data evaluation if TPs are to be included. This highlights the need for more streamlining and automation of the annotation steps. Recent developments to this end include the Patroon workflow,^53^ MetFrag,^54^ SIRIUS,^55^ and DReamS.^56^ Chromatographic separation and annotation challenges are further exacerbated when chemicals are tested as mixtures. While testing of chemicals becomes more efficient the more chemicals are assessed in one mixture, an increasing number of chemicals renders it more difficult to clearly connect precursor and TPs and, thus, increases the time spent for TP annotation while decreasing the certainty.

A recent evaluation of i*n silico* approaches for TP prediction identified limited applicability domains, data biases and mechanistic uncertainties as key limitations, consequently calling for the introduction of confidence levels for predicted TPs.^57^ Additionally, *in silico* approaches can result in the so-called “combinatorial explosion” where the number of potential TPs grows rapidly with each generation of TPs and eventually lacks discriminatory power. Methods to calculate the probability of predicted transformation reactions may counteract this by focusing the process toward the most likely products in each step, but their selectivity currently remains at 20–30% at best,^58^ which is too low for a reliable prioritization.

We envision that progress in high-throughput identification and *in silico* prediction of TPs should eventually form a positive feedback cycle: improved testing and analysis will provide more and higher quality TP data for model improvement, while advancements in *in silico* methods aid TP identification and may eventually facilitate quick and reliable TP annotation even in complex mixtures. Consequently, these approaches should not be developed independently but jointly, so that progress in one method constantly reinforces and accelerates the other.^51^

Integration of transformation products into HT-testing and into *in silico* methods can follow two fundamental strategies, which are each aligned with different goals. Approaches employed for decision making either for regulation or for early stages of chemical development may focus on persistent TPs, thus reducing the workload by limiting analysis and identification of TPs to those that remain at the end of the experiment, while congruent *in silico* prediction may focus on TPs for which no further transformation step can be predicted. Identifying precursors of persistent TPs through this approach would also support regulatory pathways for their management, such as through grouping approaches.^12^ For knowledge generation, elucidating the entire transformation pathway by repeated analysis for TPs over the whole test duration is more labor-intensive, but, in return, offers an improved mechanistic understanding of the enzymatic transformation steps leading to formation of TPs, upon which new models may be built.

## Toward a FAIR and Common Repository for Biodegradation Data

As researchers strive to generate new biodegradation data in a high-throughput manner and others invest in large-scale efforts to use the already published data as much as possible, jointly developed and publicly available templates for reporting biotransformation data and essential metadata in a transparent, electronically accessible format are urgently needed.^59^ While industry often uses the proprietary MetaPath format^60^ for this purpose, public and open formats have been missing. Harmonized requirements and guidance from journals and funding organizations will be key in establishing such open formats. Most recently, two leading journals in the field (*ES&T* and *ES&T Letters*, ACS) have updated their author guidelines to provide specific guidance on how to report pathway and kinetic information from transformation studies as well as metadata on the experimental conditions in a machine-readable format. They specifically highlight transformation tables compatible with NORMAN-SLE/PubChem for reporting transformation reactions (including their enzyme catalysts) observed under different conditions,^61^ as well as the BiotransformAtion Reporting Tool (BART) template,^62^ which also supports collection of metadata on experimental conditions and kinetic, *i.e*., half-life, information.

To move toward widespread implementation and to generate momentum, several actions are needed. First, more journals should require biotransformation data to be submitted in a FAIR format using standardized templates. Second, tools that assist in converting text and other data into a format compliant with such templates should be developed (e.g., ShinyTP^63^). Third, the developers of these templates should provide ways to submit comments and suggestions for improvements (e.g., through GitHub for BART). Last, a central curated repository for biotransformation data is required.

Toward that latter goal, the European Commission has established a “Common Data Platform” Regulation (EU 2025/2455)^64^ to support its strategy for implementing the “one chemical, one assessment” approach. The platform aims to centralize currently scattered chemicals data ensuring it follows FAIR principles. Consolidating data increases transparency, efficiency, scientific coherence and evidence-based policy making. During 2026, the Commission will adopt an implementation plan identifying data sets to be included. While we applaud this initiative, we believe that biotransformation data, in its entirety, is highly complex. Therefore, it would be beneficial to engage dedicated experts to support the successful introduction of such complex data into the common data platform and its maintenance. Due to its complexity, the possibility of a dedicated central data repository for biotransformation data is recommended to be considered. This would also provide an avenue for collaboration between academia, industry and EU regulators for advancing biotransformation data for regulatory, innovation and scientific purposes. Furthermore, the recently published OECD document no. 417 ^65^ on how to foster the use of research data for regulatory purposes provides a good starting point for the initiative.

## Let Us Go for P-NAMs

We believe that it is high time to move forward in the persistence assessment of chemicals. We see an analogy to the situation in (eco-)­toxicological assessment of chemicals some ten years ago, when it was realized that more flexible and experimentally less demanding “new approach methodologies” are needed to cope with the existing challenges in (eco-)­toxicity testing for the growing number of chemicals humans and ecosystems are exposed to.^66^ Still, there is an ongoing debate on the specificity and relevance of results provided by these NAMs, and on the level of protection that can be achieved by using them.^67^ Nevertheless, this debate has greatly intensified the scientific exchange between academia, industry and authorities on how to best generate data and accelerated the development of new test methods. This has not only fostered data production but also scientific progress in understanding mechanisms of toxicity.^68^ The ToxCast and Tox21 initiatives launched by US federal agencies in the 2000s^69,70^ are prominent examples of HT in vitro testing, which have generated results for thousands of chemicals and have been instrumental for developing models and extrapolation approaches to directly support the regulation of chemicals. We envision a similar perspective for P-NAMs but with a much stronger alignment to modern analytical chemistry methods.

Past efforts to establish HT-approaches for biodegradation testing have been impeded by the uncertain regulatory acceptance of such data, limited availability of these data sets to authorities, and the difficulty of determining how such data should be appropriately used and incorporated into an established testing hierarchy. ECHAs call for a new “middle level” test may not only encourage the scientific community to explore new HT-testing approaches but eventually become a catalyst to extend testing frameworks with such tests. Joint efforts toward the development of P-NAMs will thus pave the way in persistence assessment to cope with the challenges that exist and facilitate that transition toward safer chemicals management that society, regulators, innovators and researchers can expect.
